# Evaluation of an inducible knockout system in insect cells based on co-infection and CRISPR/Cas9

**DOI:** 10.1371/journal.pone.0289178

**Published:** 2023-07-27

**Authors:** Christina Sophie Hausjell, Miriam Klausberger, Wolfgang Ernst, Reingard Grabherr

**Affiliations:** Department of Biotechnology, Institute of Molecular Biotechnology, University of Natural Resources and Life Sciences, Vienna, Austria; East China Normal University School of Life Sciences, CHINA

## Abstract

Due to comparably high product titers and low production costs, the baculovirus/insect cell expression system is considered a versatile production platform in the biopharmaceutical industry. Its excellence in producing complex multimeric protein assemblies, including virus-like particles (VLPs), which are considered promising vaccine candidates to counter emerging viral threats, made the system even more attractive. However, the co-formation of budded baculovirus during VLP production poses a severe challenge to downstream processing. In order to reduce the amount of budded baculovirus in the expression supernatant we developed an inducible knockout system based on CRISPR/Cas9 and co-infection with two baculoviral vectors: one bringing along the Cas9 nuclease and the other one having incorporated the sequence for sgRNA expression. With our set-up high titer viruses can be generated separately, as only when both viruses infect cells simultaneously a knockout can occur. When budding essential genes *gp64* and *vp80* were targeted for knockout, we measured a reduction in baculovirus titer by over 90%. However, as a consequence, we also determined lower overall eYFP fluorescence intensity showing reduced recombinant protein production, indicating that further improvements in engineering as well as purification are required in order to ultimately minimize costs and timeframes for vaccine production utilizing the baculovirus/insect cell expression system.

## Introduction

In recent years, the baculovirus/insect cell expression system has been extensively applied for the production of numerous biotechnologically relevant compounds. Due to the possibility of inserting large gene fragments and expressing multiple foreign genes simultaneously, it is especially suitable for the production of multimeric protein vaccines, such as virus-like particles (VLPs) as well as gene therapy vectors in particular AAVs [1]. The expression platform brings along various advantageous attributes, including the capability of carrying-out mammalian-like posttranslational modifications, cultivation in serum-free suspension cultures in absence of CO_2_ and comparably high product titers and is thus, progressively used in industrial applications [2].

However, the main bottleneck within the system is the co-formation of baculovirus particle impurities during recombinant protein expression, greatly impeding the downstream process of VLP products [3,4]. Pharmaceutical products must undergo rigorous purification in order to assure their safety for human use. Although, baculoviruses are insect-specific and do not replicate in mammalian cells, they have been shown to be able to enter human cells and provoke antiviral responses in the mouse model [5–7]. Therefore, thorough purification processes need to be established in order to efficiently remove residual baculoviral impurities from the preparations. However, similar characteristics, regarding size, density and surface proteome between budded virus and enveloped VLPs, complicates their separation, frequently resulting in low process efficiency and inadequate product purity [8].

So far, several attempts have been made trying to decrease the amount of baculovirus impurities that is generated during the production process. In this respect, genes, essential for virus budding such as *gp64*, the baculovirus major envelope glycoprotein gene, or the *vp80* capsid gene have been deleted from the viral genome and thereby almost completely abolished virus budding [9,10]. Yet, in order to generate high titer virus stocks for recombinant protein production, helper cell lines that complement for the missing gene, are indispensable. Viral amplification and working stock production with complementing cell lines, however, is frequently accompanied by low efficiency and associated with overall low process productivity.

Alternative strategies for reducing virus progeny are based on RNA interference, utilizing siRNAs or dsRNAs to downregulate the expression of genes essential for budding, instead of knocking them out. Although, several of these attempts showed promising results in proof-of-concept studies, none of them are feasible for large scale production [11,12]. Others have utilized engineered insect cell lines to stably express foreign genes, omitting the need for baculovirus infection. However, product titers so far have not reached the ones obtained with the baculovirus expression vector system [13,14].

More recent strategies employ the CRISPR/Cas9 system for the knock-out/-down of viral essential genes. Bruder et al. reported a system relying on insect cell lines stably expressing Cas9 and baculoviruses engineered to support transcription of guide RNAs targeting viral essential genes, including *vp80*, *iE1* or *gp64*. With this set-up a reduction of budded baculovirus to less than 10% could be achieved [15,16]. Of note, this system is not versatile as it can only be used in combination with engineered insect cell lines, which are generally tedious to generate and are frequently instable and/or associated with low productivity [17,18]. Thus, stably engineered insect cell lines have, to the best of our knowledge, up to now not reached the biopharmaceutical industry.

To surmount these difficulties, we designed a virus-based CRISPR/Cas9 knock-out strategy in insect cells with the aim to reduce virus progeny in expression supernatants. The system can be used in any insect cell lines supporting *Ac*MNPV replication, such as *Sf*9 or HighFive^TM^, without the need for cell engineering. The platform described is based on co-infection with two engineered baculoviruses, where one encodes the Cas9 nuclease and the other provides transcription of a sgRNA specific for a baculovirus essential gene. In this manner high titer virus stocks can be separately amplified and only upon co-infection of cells, gene editing can occur, production of budding essential proteins and viral budding is impaired. As targets we selected *gp64* and *vp80*, which have been shown before to be indispensable for efficient virus budding [19,20]. With our experimental set-up we achieved a reduction in viral titer by more than 90% compared to the control. However, we also measured lower levels of overall eYFP fluorescence intensity, which served as recombinant model protein.

## Methods

### Cell culture

*Sf*9 cells (ATCC-CRL-1711) were maintained in serum-free HyClone SFM4 Insect medium (Cytiva) with L-Glutamine supplemented with 0.1% Kolliphor P188 (Sigma Aldrich). Cells, tested negative for mycoplasma, were cultured in Erlenmeyer shaking flasks at 110 rpm and 27°C.

### Molecular cloning and virus generation

A pACEBac1gp64 promoter transfer plasmid was generated by exchanging the polyhedrin (polh) promoter on the pACEBac1 acceptor vector (Geneva Biotech) against the *Ac*MNPV gp64 promoter. Briefly, pACEBac1 was digested with *Cla*I and *BamH*I restriction enzymes and the gp64 promoter amplicon—amplified from an isolated MultiBac bacmid–was digested with the same enzymes and ligated into the prepared vector. The Cas9 coding sequence (codon-optimized for expression in Drosophila) was PCR-amplified from the plasmid pBS-Hsp70-Cas9 (#46294, Addgene), digested with *EcoR*I and *Hind*III and was cloned into pACEBac1gp64 interjacent the gp64 promoter and SV40 terminator, yielding pACEBac1gp64-Cas9. The Cas9 sequence includes an N-terminal 3xFlag-tag (DYKDHDGDYKDHDIDYKDDDDK) and SV40 nuclear localisation signal (PKKKRKV) and a C-terminal nucleoplasmin nuclear localisation signal (KRPAATKKAGQAKKKK).

A pIDK donor vector (Geneva Biotech) harbouring the RNAIII U6-2 promoter was generated [21]. Briefly, the vector was digested with *Spe*I and *Xho*I to remove the *Ac*MNPV p10 promoter. The U6-2 promoter was PCR-amplified from genomic DNA isolated from cultured *Sf*9 cells with primers [ATGATGATGACTAGTAATATACATACACATTTTATTGAAAATCAC; ATGATGATGCTCGAGACTTGCCAAGGCAAA], *Spe*I/*Xho*I-digested and was ligated into the digested pIDK vector to yield pIDK-*Sf*9U6-2. Then further, a pACEBac1-*Sf*9U6-2 vector was generated by exchanging the polh promoter with the *Sf*9U6-2 promoter. Briefly pACEBac1 was digested with *Cla*I and *Xba*I, the U6-2 promoter was amplified from pIDK-*Sf*9U6-2, the amplicon was digested with the same enzymes and ligated with the digested vector, resulting in pACEBac1-*Sf*9U6-2.

SgRNAs, targeting *Ac*MNPV *gp64* or *vp80*, were designed with the Benchling CRISPR Guide RNA design tool (https://www.benchling.com). For each viral gene two sgRNA sequences were selected based on on-target score and off-target score setting the *Spodoptera frugiperda* genome as off-target genome (Table 1). SgRNA sequences were ordered as synthetic gene fragments (gblocks) from Integrated DNA Technologies (IDT). SgRNA sequences were PCR-amplified, digested with *Xba*I and *Avr*II and ligated into pACEBac1-*Sf*9U6-2, previously digested with the same enzymes. The empty pACEBac1-*Sf*9U6-2 vector served as control in all experiments. All generated plasmids were sequenced by sanger sequencing.

**Table 1:** Protospacer sequences of sgRNAs (5’-3’).

sgRNA	Protospacer sequence (5’-3’)	PAM	Strand
sgRNA gp64 1	GGTTAGAGCCAAGTACACAG	AGG	Template
sgRNA gp64 2	GGAAATCACCATCGTGGAGA	CGG	Template
sgRNA vp80 1	GGGTCGCTGGATGTTACCCG	CGG	Nontemplate
sgRNA vp80 2	GGACACGTTAGAGGTAATGT	TGG	Template

Recombinant baculoviruses (rBVs) were generated by transforming pACEBac1 plasmid derivatives into DH10EMBacY cells (Geneva Biotec) that carry an *eYFP* expression cassette under control of the polh promoter. Bacmid-DNA was isolated and transfected into *Sf*9 cells using FuGene HD transfection reagent (Promega) according to the manufacturer’s instructions. Recombinant baculoviruses were amplified to P2 stocks in *Sf*9 cells. This yielded one rBV that carried a Cas9 expression cassette (*Ac*-Cas9), four rBVs that enabled transcription of two gp64 or vp80 sgRNAs, respectively (*Ac*-sgRNA gp64 1; *Ac*-sgRNA gp64 2; *Ac*-sgRNA vp80 1; *Ac*-sgRNA vp80 2) and one pACEBac-*Sf*9U6-2 virus that served as control (*Ac*-empty). Viral titers were evaluated by a TCID_50_ endpoint dilution assay.

### Co-infections

Co-infections were carried out in 6-well tissue culture plates. Cells were seeded at a density of 1x10^6^ cells/well in cell culture medium and were co-infected with *Ac*-Cas9 and one of the four *Ac*-sgRNA viruses or *Ac*-empty, respectively. Appropriate volumes of virus solutions for co-infections were premixed in 1 mL medium to reach a final multiplicity of infection (MOI) of 5 plaque forming units (PFU)/cell for *Ac*-sgRNA gp64 1, *Ac*-sgRNA gp64 2, *Ac*-sgRNA vp80 1, *Ac*-sgRNA vp80 2 or *Ac*-empty and a final MOI of 1 or 5 PFU/cell for *Ac*-Cas9. The initial medium was removed and premixed virus solutions were added dropwise to the cells. After 1 h of incubation at 27°C, the virus inoculum was removed, cells were washed twice with medium, before 1 mL fresh medium was added dropwise to each well and plates incubated at 27°C for 72 h. All experiments were performed in three biological replicates.

### Endpoint dilution assay

Baculovirus titers were determined by TCID_50_. Briefly, 100 μL *Sf*9 cells/well, at a density of 4x10^5^ cells/mL were seeded in 96-well plates. Virus suspensions were 5-fold serially diluted and 20 μL of each dilution was added to the cells in 8 replicates. After 5 days of incubation at 27°C, fluorescence was evaluated with a Leica Fluorescence microscope. TCID_50_ units were calculated using a modified equation from Reed an Muench and converted to PFU/mL using a Poisson distribution-derived factor [22]. Statistical significance was determined by one-way ANOVA.

### Plaque assay

Additionally, viral titers were evaluated by standard plaque assay. Briefly, *Sf*9 cells were seeded in 6-well plates at a density of 1x10^6^ cells/well. Supernatants from co-infections were 10-fold serially diluted in duplicates and 500 μL of each virus dilution was added to the cells. Plates were incubated for 1 h at 27°C, before the viral inoculum was removed and wells were overlaid with 2 mL of cell culture medium supplemented with 1% low melting temperature agarose (Sigma-Aldrich), 10% FBS (Gibco) and 1 x Antibiotic-antimycotic mix (Gibco). After incubation for 6 days at 27°C in a humid environment, plaque assays were stained with 1 mg/mL MTT (Sigma-Aldrich), plaques were counted, and the titer determined as PFU/mL. One-way ANOVA was applied to evaluate statistical significance.

### Flow cytometry

Three days post co-infection, cells were pelleted (500 x g, 5 min), washed in 800 μL PBS, recentrifuged at 500 x g for 5 min, and resuspended in 500 μL PBS. eYFP fluorescence intensity was measured utilizing a CytoFLEX S flow cytometer (Beckman) and data analysis was performed with the Kaluza 2.1 software (Beckman). The overall eYFP fluorescence intensity (sum of intensity) was calculated by multiplying the number of fluorescent cells with the arithmetic mean of the fluorescence intensity.

### qPCR

Total DNA was extracted from co-infected cells utilizing the monarch genomic DNA isolation kit (Neb) according to the manufacturer’s instructions. qPCR was performed with the Luna Universal qPCR Master Mix (Neb) according to the protocol provided by the manufacturer. For quantification of relative *eYFP* DNA levels gene specific primers were used [GGCACAAGCTGGAGTACAAC; AGTTCACCTTGATGCCGTTC] [23]. For quantification of relative *vp39* DNA levels gene specific primers were designed with the Genescript Real-time PCR (TaqMan) Primer and Probes Design Tool [ACTTGCAAATCGACACGGAGGA; AGCTCACGTGTGTCGCCTTC]. *Ecdysoneless* (*ECD)*, previously proven suitable as reference gene during baculovirus infection of *Spodoptera frugiperda* cells, served as internal reference [GCCGTATCCAAAGGATGACA; TGGTGACGGCCAAAGGAA] [24]. 20 μL qPCR reactions consisted of 10 μL Luna Universal qPCR Master Mix, 0.5 μL forward primer (10 μM), 0.5 μL reverse primer (10 μM), 1 ng DNA template and nuclease-free water (to 20 μL). qPCR was conducted on a C1000 Themal Clycler with CFX96 Real-Time PCR Detection System (Bio-Rad) under the following cycling conditions: initial denaturation at 95°C for 60 sec, followed by 40 cycles of denaturation at 95°C for 15 sec, elongation at 60°C for 30 sec and a subsequent plate read. Melt curve analysis were performed to verify specific amplification. Relative levels of *eYFP* and *vp39* DNA were determined with the 2^ΔΔcq^ method [25]. Statistical significance was evaluated via Student’s *t*-test.

## Results

### Set-up of the inducible knockout system

**Fig 1:**
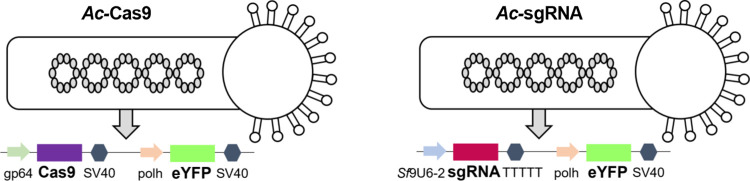
Set-up of the co-infection based inducible knockout system. The inducible knockout system is based on co-infection with two viral vectors, the first one carrying the sequence for the expression of the Cas9 nuclease under control of the baculoviral gp64 promoter and the second one carrying the sequence for the expression of the sgRNA under control of an *Sf*9-U6-promoter. Additionally, all viral vectors bring along a sequence for expressing the model protein eYFP under control of the very late baculovirus polyhedrin (polh) promoter.

Generating and maintaining stable insect cell lines can be demanding and approaches for reducing baculovirus particle formation that rely on stably engineered helper cell lines are often accompanied by loss of productivity [9,10]. Therefore, we develop a fast and versatile baculovirus-based knockout system aimed at impeding baculovirus formation and thereby reducing the viral burden in expression supernatants. Our approach relies on co-infecting insect cells with two distinct recombinant baculovirus vectors (Fig 1), one of which comprising a Cas9 expression cassette containing the *Ac*MNPV gp64 promoter (*Ac*-Cas9) and the second vector allowing for the transcription of sgRNAs under control of a constitutive *Sf*9 polymerase III promoter (*Ac*-sgRNA). For expression of Cas9 the gp64 promoter was chosen, since as compared to expression from a very late baculovirus promoter such as the polh promoter, it leads to earlier availability of the Cas9 nuclease at sufficiently high levels [26]. As targets we selected *gp64* and *vp80*, which have both been shown to be required for efficient baculovirus budding [19,20]. GP64 is the baculovirus major envelope glycoprotein and essential for cell entry as well as virus egress [27,28]. Its expression peaks twice, moderately strong early and stronger late during the viral life cycle [29]. The baculovirus minor capsid protein VP80 is transcribed late in virus replication and was shown to be indispensable for migration of nucleocapsids to the cellular periphery and consequently budded virus propagation [20]. For knockout, we designed and tested two sgRNAs for each gene. Our system, employing a co-infection strategy brings along the advantage, that rBVs may be individually amplified to high-titer virus stocks and the CRISPR/Cas9 machinery only comes into action once cells are infected with both viruses simultaneously.

### Co-infection impacts baculovirus replication

**Fig 2:**
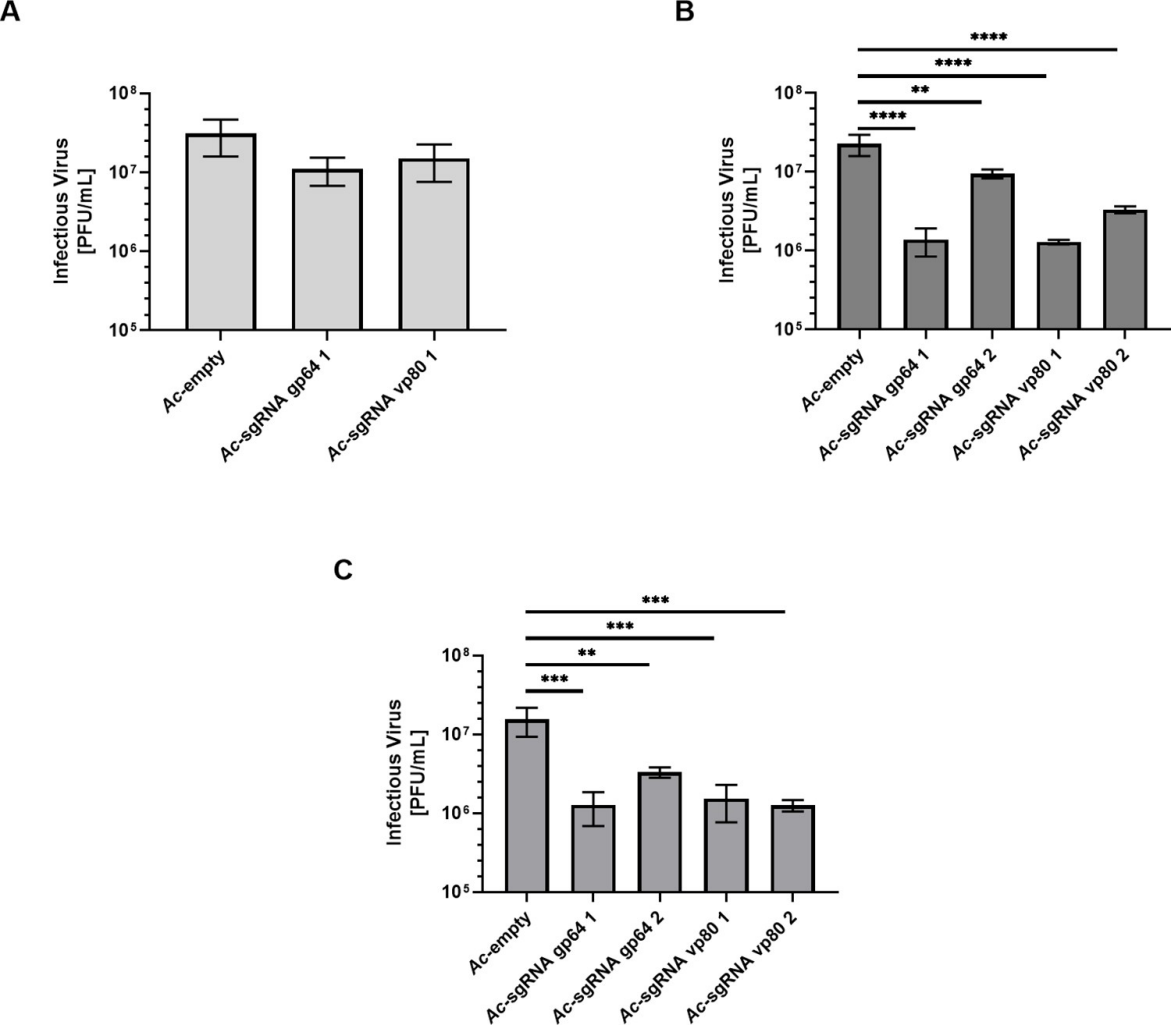
Baculovirus titers after co-infection. (A) Infectious virus titer of supernatants co-infected with *Ac*-sgRNA or *Ac*-empty at MOI 5 and *Ac*-Cas9 at MOI 1, determined via TCID_50_ (B) Infectious virus titer of supernatants co-infected with *Ac*-sgRNA or *Ac*-empty and *Ac*-Cas9 both at MOI 5, determined via TCID_50_ (C) Infectious virus titer of supernatants co-infected with *Ac*-sgRNA or *Ac*-empty and *Ac*-Cas9 both at MOI 5, measured by plaque assay. ** p < 0.01; *** p < 0.001; **** p < 0.0001.

First, we evaluated the efficiency of our system to knockout baculoviral genes and thereby reduce virus budding. In an initial screening experiment, *Sf*9 cells were co-infected with the two baculoviruses at two different MOI-combinations, but at constant volumes: While *Ac*-sgRNA gp64 1, *Ac*- sgRNA vp80 1 or the control virus *Ac*-empty were added at MOI 5 in all experiments, cells were co-infected with *Ac*-Cas9 at MOI 1 or MOI 5, respectively. The viral titers were determined 72 h post infection (hpi) via TCID_50_. As shown in Fig 2A, viral titers were comparable (1.1x10^7^-3.1x10^7^ PFU/mL) when adding the viruses at the MOI combination of 5–1. Yet, when adding both viruses at MOI 5 we observed a significant titer reduction (about 90%) after co-infection with all *Ac*-Cas9 –*Ac*-sgRNA combinations as compared to the control (Fig 2B). Thus, for all upcoming experiments, we only considered the MOI combination of 5–5.

We then further included *Ac*-sgRNA gp64 2 and *Ac*-sgRNA vp80 2 into our analyses. Co-infection with *Ac*-sgRNA vp80 2 also significantly lowered the viral titer by around 85%, while the reduction with *Ac*-sgRNA gp64 2 was the least pronounced (by ~60%) (Fig 2B). Plaque assays were performed to confirm these results by an ortholog method. There, *Ac*-sgRNA gp64 1, *Ac*-sgRNA vp80 1 and *Ac*-sgRNA vp80 2 proved to be highly efficient in reducing baculovirus budding, resulting in a baculovirus titer reduction of over 90%. The co-infection with *Ac*-sgRNA gp64 2 showed again the lowest efficiency, with a decline by around 80% (Fig 2C).

### Co-infection influences recombinant protein production

**Fig 3:**
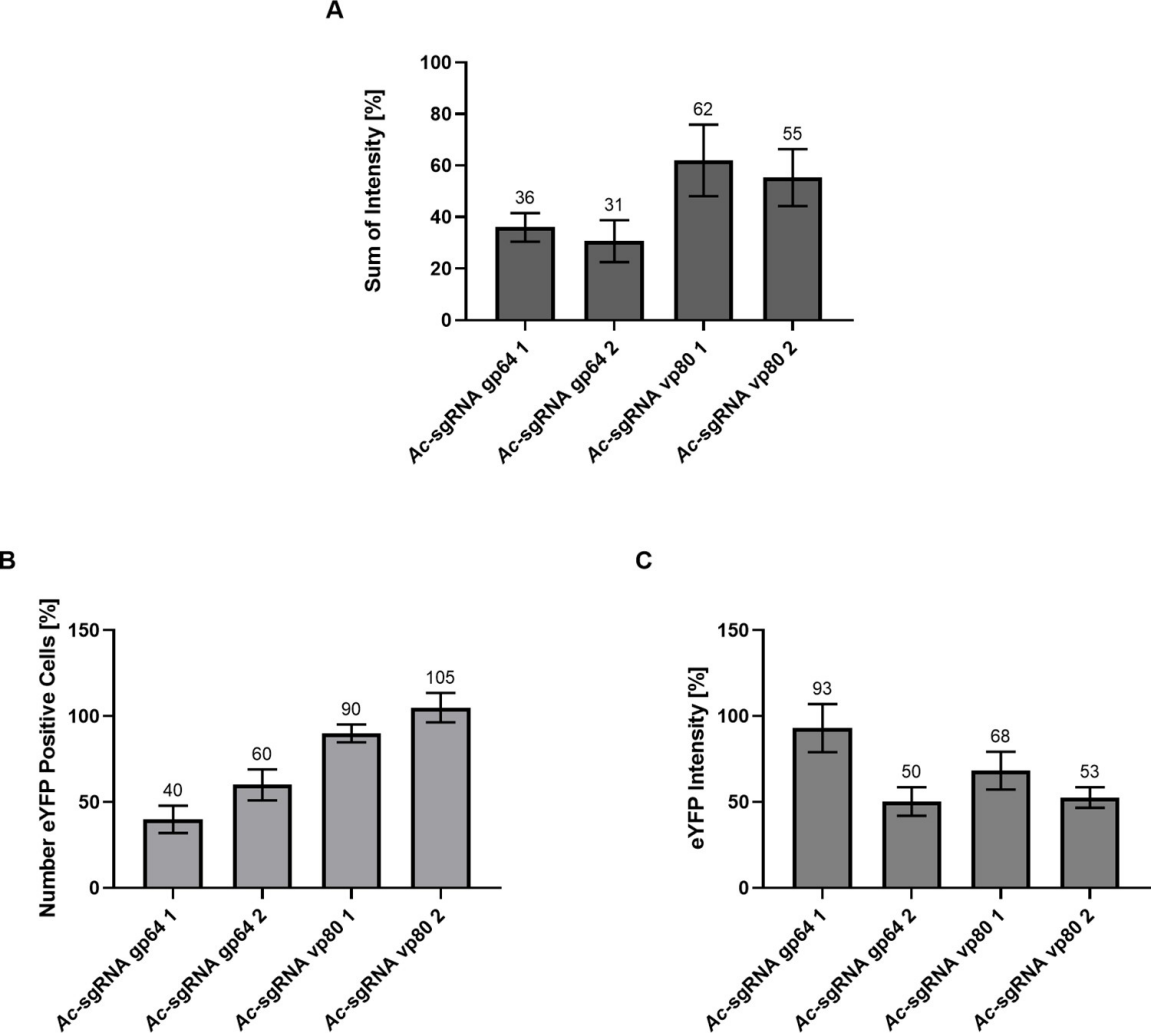
eYFP fluorescence intensity measured by flow cytometry. (A) Overall eYFP fluorescence intensity (sum of intensity) of cells co-infected with *Ac*-sgRNA and *Ac*-Cas9 compared to a co-infection with the control virus (*Ac*-empty and *Ac*-Cas9) (B) Number of eYFP positive cells after co-infection with *Ac*-sgRNA and *Ac*-Cas9 compared to a co-infection with the control virus (*Ac*-empty and *Ac*-Cas9) (C) eYFP fluorescence intensity of cells co-infected with *Ac*-sgRNA and *Ac*-Cas9 compared to a co-infection with the control virus (*Ac*-empty and *Ac*-Cas9).

All recombinant baculovirus used for co-infection additionally contained an expression cassette for eYFP under control of the very late baculoviral polyhedrin promoter, usually exploited for recombinant protein production (Fig 1). Therefore, we next analysed whether the knockout and titer reduction would influence very late gene expression and thus, recombinant protein production, using eYFP as a model protein. *Sf*9 cells co-infected with *Ac*-Cas9 and one of the *Ac*-sgRNA viruses or the control virus *Ac*-empty were analysed for their overall eYFP fluorescence intensity via flow cytometry 72 hpi. As shown in Fig 3A, for all co-infections of *Ac*-Cas9 with a rBV supporting sgRNA transcription, we saw a reduction in the sum of fluorescence intensity compared to the co-infection with the control virus. The reduction in overall fluorescence intensity was most pronounced for co-infections with the two viruses encoding the baculovirus gp64 sgRNAs (-60-70%). In contrast, when *vp80* was targeted, the overall eYFP fluorescence intensity was only reduced by about 40–50%. When splitting up the signal of overall fluorescence intensity into percentage of eYFP positive cells and eYFP intensity, we saw different effects for the different knockout setups. For *Ac*-sgRNA gp64 1 it was the number of eYFP positive cells that was greatly reduced, while for *Ac*-sgRNA gp64 2 both number and signal intensity were decreased. The co-infections with *Ac*-sgRNA vp80 1 and *Ac*-sgRNA vp80 2 on the other hand, led mainly to a reduction in fluorescence intensity, while the number of eYFP positive cells, remained roughly the same as the one of the co-infection with the control (Fig 3B and 3C).

### qPCR reveals lower levels of viral DNA in cells

**Fig 4:**
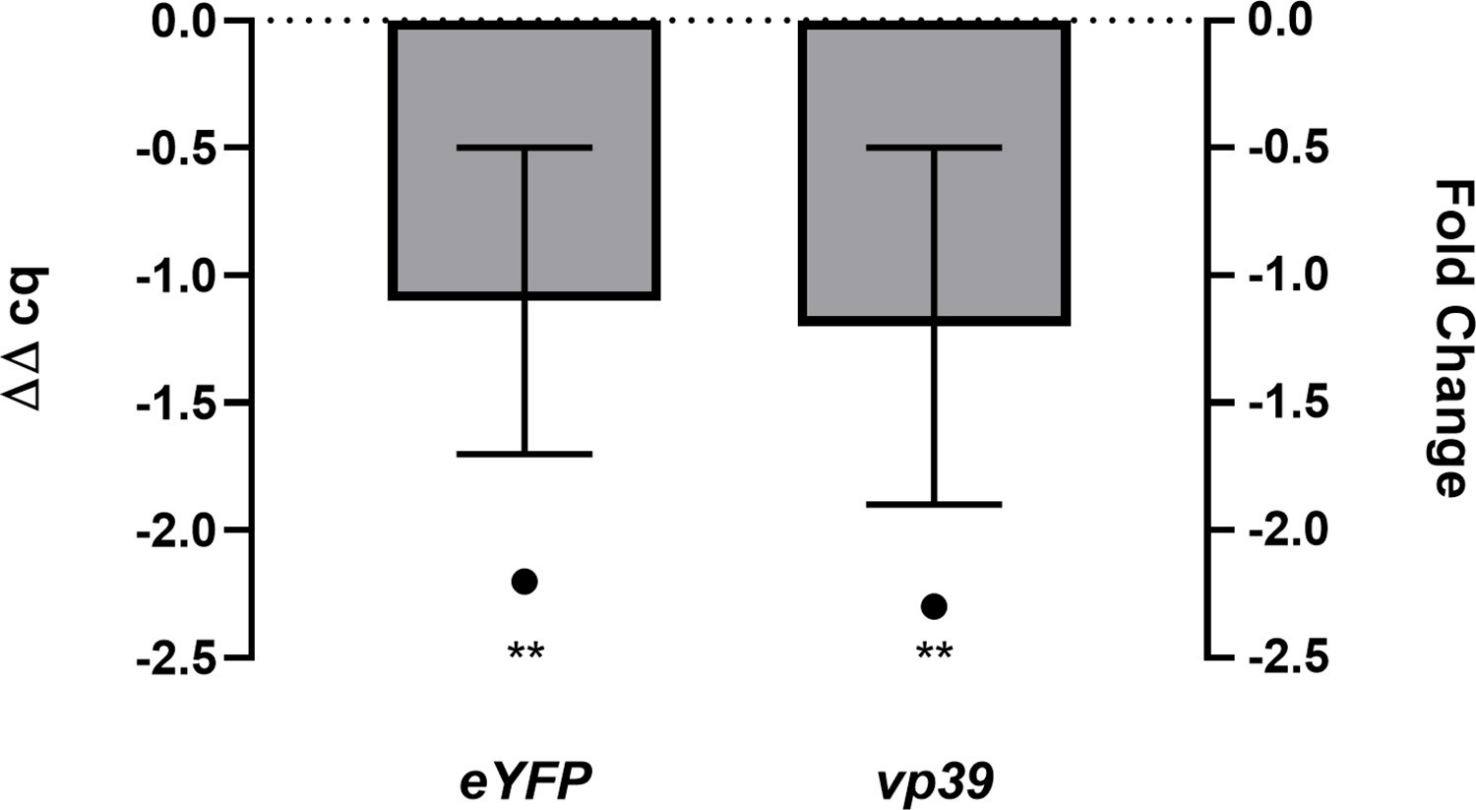
Relative viral DNA levels determined by qPCR. ΔΔcq and corresponding fold change values of *eYFP* DNA and *vp39* DNA after co-infection with *Ac*-sgRNA vp80 1 and *Ac*-Cas9 compared to co-infection with *Ac*-empty and *Ac*-Cas9. ** p < 0.01.

When co-infecting *Sf*9 cells we determined a reduction in baculovirus titer, but also in very late gene expression. In order to investigate whether the observed effects might be caused by lower levels of viral DNA inside cells, we determined the relative DNA levels of *eYFP* and baculoviral *vp39* capsid gene. *Sf*9 cells were therefore co-infected with *Ac*-Cas9 and *Ac*-sgRNA vp80 1 or *Ac*-empty, both at MOI 5. We chose *Ac*-sgRNA vp80 1 for the experiment, since it looked the most promising from previous results (highest titer reduction, lowest eYFP signal loss). Cellular DNA was isolated 72 hpi and relative DNA levels were determined on basis of the *eYFP* and *vp39* gene, respectively by qPCR utilizing the 2^ΔΔcq^ method. Fig 4 shows the ΔΔcq and matching fold change values resulting from a comparison of Δcq values from the co-infection with the control virus *Ac*-empty (ΔΔcq = 0; fold change = -1). Co-infection with *Ac*-sgRNA vp80 1 lead to a fold change of -2.2 of *eYFP* DNA and -2.3 *vp39* DNA levels respectively, confirming a reduction of viral DNA 72 h post co-infection with the virus supporting sgRNA expression.

## Discussion

In this study we developed an inducible knockout platform in insect cells, based on CRISPR/Cas9 and co-infection, utilizing solely baculoviruses as the carrier of recombinant sequences, omitting the need for cumbersome cell engineering. We showed that a co-infection where one virus brings along the sequence for the expression of the Cas9 nuclease and the other one the sequence of the sgRNA can reduce baculovirus titers by over 90%, when either *gp64* or *vp80* are targeted for knockout. However, the phenomenon only comes into effect once both viruses are added at high MOI (MOI 5). Those data are consistent with previous studies, showing that during infections with multiple viral vectors utilizing the right MOI combination is crucial in order to reach cells with all virus constructs [30,31].

In total, we tested four different sgRNAs targeting either *gp64* or *vp80*, which had varying efficiencies regarding titer reduction, supporting an important role of sgRNA design [32].

We further evaluated the effect of co-infection and titer reduction on recombinant protein production utilizing eYFP as a model protein. We saw a marked reduction in overall eYFP fluorescence intensity (by ~60–70%) when *gp64* was targeted for silencing. When *vp80* was targeted however, the reduction was to a much lower extent (by ~40–50%). Knockout of *gp64* and *vp80* in previous studies did not influence very late gene expression [9,10,16]. When performing qPCR, we determined lower levels of *eYFP* and *vp39* DNA upon co-infection of cells with the *Ac*-sgRNA vp80 1 virus compared to the control co-infection with *Ac*-empty. Whether this reduction in genome copy number is due to a hampered secondary infection or a degradation of baculovirus genomes after Cas9 cleavage can only be speculated. Statistically, when infected at a high MOI such as 5 PFU/cell, essentially all cells should be infected synchronously. However, the reduction in virus propagation cannot rule out a decrease in secondary infections. Nevertheless, lower gene dosage and protein expression is not expected to be caused by fewer secondary infections. Degradation of linearized baculovirus DNA can also not be excluded. Although, CRISPR/Cas9 is nowadays a well-established tool for genome editing in various organisms, it has only been adapted for application within the baculovirus/insect cell expression system recently [33,34]. In eukaryotic cells, usually once DNA is cut by the CRISPR/Cas9 machinery it is repaired by cellular repair mechanisms, that however are error-prone often resulting in insertions or deletions and ultimately loss of function mutations [35]. In our study for the first time, both, Cas9 and sgRNA are delivered via baculovirus infection. The host machinery is shut down shortly after virus infection and whether the baculovirus encoded DNA polymerase is sufficient to repair after Cas9-cleavage is not known [36]. Linear baculovirus DNA within cells is fairly unstable and if repair mechanisms are impaired by virus infection or not available, less viral DNA is present resulting in reduced levels of baculovirus in the supernatant, but also lower levels of *eYFP* that can be transcribed and translated into protein and thus, less fluorescence signal is being detected. However, elucidation of the mechanism of Cas9 cleavage and repair of baculovirus DNA will help to clarify. Further experiments would be needed to explain the observed difference in the degree of signal reduction between targeting *gp64* and *vp80*. In the case of *gp64* there was a large population of eYFP-negative cells, however we do not think that this is due to technical limitations or addition of an inadequate amount of virus. Although, nucleotide sequences of *gp64* and *eYFP* do not show levels of similarity, off-target effect of sgRNAs cannot be ruled out, as well as differences in binding efficiency of the sgRNAs.

Overall, we here describe a novel inducible knockout system in insect cells, that has the potential to markedly reduce the burden of co-formed baculovirus particles in expression supernatants, however effects recombinant protein production as well. Previous attempts in reducing viral particle formation, especially deletion of budding-essential genes *gp64* and *vp80* from the virus genome led to an even greater reduction in baculovirus titer as compared to our approach (~90% vs single cell infections). However, the need for helper cell lines to generate high titer viruses for production, was accompanied by a great loss in productivity [9,10,28]. Bruder et al., recently reported a system based on CRISPR/Cas9 as well, where they showed a titer reduction by more than 90%, without effecting recombinant protein yield. However, with their platform again a considerable amount of budded virus is still present, similar to what we have observed in our study [16]. The reason behind this might be that when the time point is reached where enough sgRNA, and in our case Cas9 are present and may act in the system, already sufficient viral proteins have been generated to drive virus budding. Additionally, the amount of generated Cas9 and sgRNA might not be enough to meet the amount of virus DNA that is produced during virus replication; hence a certain quantity of budded virus is still found in the supernatant.

While our approach led to a sufficiently reduced baculovirus titer that would facilitate downstream processing, it is accompanied by a loss in productivity, rendering application in industry uneconomic. Despite the vast number of approaches that have been investigated, none has succeeded in completely diminishing baculovirus budding without interfering with productivity. Future studies and innovative engineering strategies together with advances in separation techniques will be necessary to lower costs and timeframes and further improve recombinant protein production utilizing the baculovirus/insect cell expression system.

### Conclusion

In this study we describe a novel inducible knockout platform in insect cells. Our system is based on co-infection with two viral vectors, one carrying the Cas9-sequence and the other one bringing along the sgRNA sequence. Knockout can only occur once cells are co-infected with both viruses and therefore, high titer viruses can be raised as long as both constructs are kept apart from each other. With our system, when targeting baculovirus *gp64* and *vp80*, we saw a titer reduction of 58–94%. However, we also observed lower levels of recombinant protein as shown by the model protein eYFP, where the overall eYFP fluorescence intensity was diminished by 40–70%. To conclude, our platform enables baculoviral titer reductions of over 90% without the need of helper cells lines, however, it does not allow for an unimpaired recombinant protein production, that is necessary to facilitate downstream processing at large scale. Since none of the proposed strategies up to now does, new innovative approaches will be necessary, to ultimately further improve biopharmaceutical production employing the baculovirus insect cell expression system.

## Supporting information

S1 File Dataset. (XLSX)
